# Clearance of micronutrients during continuous renal replacement therapy

**DOI:** 10.1186/s13054-020-03347-x

**Published:** 2020-10-19

**Authors:** Nuttha Lumlertgul, Danielle E. Bear, Marlies Ostermann

**Affiliations:** 1 Department of Critical Care, King’s College London, Guy’s & St Thomas’ NHS Foundation Trust, 249 Westminster Bridge Road, London, SE1 7EH UK; 2 Division of Nephrology, Department of Internal Medicine and Excellence Center in Critical Care Nephrology, King Chulalongkorn Memorial Hospital, 1873 Rama IV Road, Bangkok, 10330 Thailand; 3 Research Unit in Critical Care Nephrology, Chulalongkorn University, 1873 Rama IV Road, Bangkok, 10330 Thailand; 4 Department of Nutrition and Dietetics, Guy’s & St Thomas’ NHS Foundation Trust, 249 Westminster Bridge Road, London, SE1 7EH UK

Malnutrition is common in critically ill patients with acute kidney injury (AKI), especially if renal replacement therapy (RRT) is needed. There are several potential explanations, including nutrient losses during RRT. Although previous studies confirmed that micronutrients were detectable in effluent fluid [1–4], daily losses have not been formally quantified. In addition, information about the transport characteristics of individual micronutrients during RRT is lacking.

We recently measured serial plasma concentrations of vitamins, trace elements, carnitine and 22 amino acids (AAs) for up to six consecutive days in 55 critically ill adult patients with severe AKI [5]. The main findings were that patients treated with continuous renal replacement therapy (CRRT) had significantly lower plasma concentrations of citrulline, glutamic acid and carnitine at 24 h after enrolment and significantly lower plasma glutamic acid concentrations at day 6 compared to non-CRRT patients. In > 30% of CRRT patients, the plasma nutrient concentrations of zinc, iron, selenium, vitamin D_3_, vitamin C, tryptophan, taurine, histidine and hydroxyproline were below the reference range throughout the 6-day period.

Loss of nutrients into the effluent fluid depends on their plasma concentration (*C*_pl_), sieving coefficient (SC) and dose and duration of RRT. The SC describes a solute’s permeability across the dialysis membrane and depends on molecular size, electric charge (Donnan equilibrium), protein binding, volume of distribution, filter porosity, contact time and adsorption to the membrane. It is calculated from the ratio of effluent to plasma solute concentration ($\frac{{\left[ {C_{{{\text{eff}}}} } \right]}}{{\left[ {C_{{{\text{pl}}}} } \right]}}$). A SC less than one represents a mass transfer process where the concentrations have not equilibrated.

Here, we report the SCs and daily total losses of AAs, vitamins, trace elements and carnitine of all 33 CRRT patients recruited to the study mentioned above [5]. Total daily loss was calculated as *C*_pl_ × SC × effluent volume per day. In addition, we estimated total losses for standard CRRT at 25 ml/kg/h for 24 h. Table 1 lists the SCs for all important nutrients and average daily losses during CRRT for up to 6 days. The key findings are:

1. Despite small molecular weights, the SCs of nutrients varied.
2. The SC of all but 2 AAs was below 1 indicating incomplete equilibration during RRT. Hydroxyproline had the highest SC (6.63). The exact reasons for SCs greater than 1 are not clear and warrant further investigations.
3. The absence of small-molecule water-soluble vitamin B1, B6 and B12 in the effluent was unexpected. However, we note that Oh et al. reported similar findings and speculated that dilution by the effluent, conversion to alternative metabolites not discriminated by mass spectrometry or adsorption by the hemofilter may have contributed [1].
4. The high daily losses of carnitine, vitamin C and trace elements in the effluent were consistent with reports in the literature [2–4].

**Table 1:** Mean sieving coefficient and daily loss of amino acids, vitamins and trace elements

Nutrient	Molecular weight [g/mol]	Mean SC^a^ ± SE	95% CI	Daily loss^b^ [mg]	Standardized daily loss^c^ [mg]
Alanine	89.1	1.02 ± 0.03	0.95–1.09	1102.3 ± 98.4	603.7 ± 37.2
Arginine	174.2	0.99 ± 0.04	0.91–1.07	427.9 ± 42.4	237.6 ± 16.3
Aspartic acid	133.1	0.82 ± 0.07	0.67–0.97	32.9 ± 2.3	20.3 ± 1.9
Citrulline	175.2	0.93 ± 0.05	0.82–1.03	756.0 ± 45.3*	439.4 ± 24.9*
Glutamic acid	147.1	0.53 ± 0.03	0.47–0.60	208.71 ± 17.3	118.4 ± 7.4
Glutamine	146.2	0.96 ± 0.03	0.90–1.01	2525.9 ± 172.8	1397.3 ± 68.1
Glycine	75.1	0.89 ± 0.03	0.82–0.96	558.1 ± 39.8	317.4 ± 20.1
Histidine	155.2	0.83 ± 0.02	0.78–0.87	387.9 ± 27.7	216.4 ± 12.1
Hydroxyproline	131.1	6.63 ± 0.83	4.94–8.31	224.7 ± 28.6	131.3 ± 18.5
Isoleucine	131.2	0.94 ± 0.02	0.89–0.99	373.9 ± 35.2	206.9 ± 13.1
Leucine	131.2	0.81 ± 0.02	0.76–0.86	592.5 ± 57.8	330.2 ± 21.9
Lysine	146.2	0.88 ± 0.03	0.83–0.94	968.1 ± 90.3	535.4 ± 38.6
Methionine	149.2	0.90 ± 0.03	0.83–0.97	182.5 ± 19.4	100.0 ± 8.1
Ornithine	132.2	0.70 ± 0.02	0.66–0.74	291.1 ± 28.5	161.4 ± 11.2
Phenylalanine	165.2	0.91 ± 0.03	0.85–0.96	626.1 ± 57.8	349.6 ± 25.1
Proline	115.1	0.75 ± 0.02	0.71–0.79	558.4 ± 47.7	308.0 ± 21.4
Serine	105.1	0.96 ± 0.04	0.88–1.04	339.3 ± 24.4	196.8 ± 9.9
Taurine	125.2	0.77 ± 0.08	0.62–0.93	124.4 ± 2.2	71.1 ± 12
Threonine	119.1	1.00 ± 0.03	0.95–1.06	496.8 ± 43.7	276.4 ± 20.0
Tryptophan	204.2	0.55 ± 0.03	0.49–0.61	128.1 ± 11.9	72.8 ± 4.9
Tyrosine	181.2	0.96 ± 0.02	0.91–1.01	554.5 ± 51.9	307.3 ± 20.9
Valine	117.1	0.88 ± 0.02	0.84–0.93	895.5 ± 81.7	499.4 ± 29.6
Carnitine	161.2	0.92 ± 0.04	0.83–1.01	1698.0 ± 134.7*	981.9 ± 75.8*
Vitamin B1	265.4	UD	UD	UD	UD
Vitamin B6	169.2	UD	UD	UD	UD
Vitamin B12	1355.4	UD	UD	UD	UD
Vitamin C	176.1	0.83 ± 0.07	0.69–0.98	100.5 ± 15.3	59.0 ± 9.2
Vitamin D2	397	UD	UD	UD	UD
Vitamin D3	384.6	UD	UD	UD	UD
Copper	63.6	0.009 ± 0.002	0.006–0.013	0.33 ± 0.05	0.20 ± 0.03
Iron	55.8	0.02 ± 0.01	0–0.04	0.07 ± 0.02	0.04 ± 0.09
Folate	441.4	0.51 ± 0.03	0.44–0.58	59.9 ± 11.7**	35.3 ± 6.9**
Selenium	79.0	0.036 ± 0.02	0–0.08	0.04 ± 0.01	0.04 ± 0.02
Zinc	65.4	0.10 ± 0.07	0–0.24	0.67 ± 0.20	0.64 ± 0.32

^*^µmol/day **µg/day

*SC* sieving coefficient, *SE* standard error, *CI* confidence interval, *UD* undetected

^a^SC was calculated as $\frac{{\left[ {C_{{{\text{eff}}}} } \right]}}{{\left[ {C_{{{\text{pl}}}} } \right]}}$, where *C*_eff_ is effluent concentration and *C*_pl_ is plasma concentration

^b^Daily loss (mg) was calculated by *C*_pl_ × SC × effluent volume per 24 h

^c^Standardized daily loss (mg) was estimated for CRRT dose 25 mL/kg/h for 24 h

Nutrition in AKI is an under-researched area, and the role of routine micronutrient supplementation in patients receiving CRRT is unknown [6]. Our data support future studies in this field. We acknowledge some limitations. First, we measured nutrient concentrations but did not investigate any relevant metabolic pathways and therefore cannot comment on the clinical impact of nutrient losses. Second, we only included patients who were established on full enteral nutrition and received CRRT for up to 6 days. Whether the results also apply to patients receiving parenteral nutrition or CRRT for longer periods is unclear. Finally, we are unable to make recommendations for nutritional support in clinical practice but suggest that intervention studies are urgently required.

## Data Availability

The datasets used and/or analyzed during the current study are available from the corresponding author on reasonable request.
